# Calibration-free picosecond LIPS for quantifying heavy metals in soils near Egyptian industrial sites

**DOI:** 10.1038/s41598-025-04395-5

**Published:** 2025-06-06

**Authors:** Mohamed El-Saeed, Walid Tawfik, Ahmed A. I. Khalil, Manal Mubarak, Mohamed Fikry

**Affiliations:** 1 Laser Sciences and Interactions Department, National Institute of Laser Enhanced Sciences (NILES), Cairo University, Giza, 12613 Egypt; 2 Department of Laser Applications in Metrology, Photochemistry and Agriculture, National Institute of Laser Enhanced Sciences (NILES), Cairo University, Giza, 12613 Egypt; 3 Soil and Water Department, Faculty of Agriculture, Ain Shams University, Cairo, 11241 Egypt; 4 Ultrafast Picosecond Laser Lab, Physics Department, Faculty of Science, Cairo University, Giza, 12613 Egypt; 5 Egypt Nanotechnology Center (EGNC), Faculty of Nanotechnology for Postgraduate Studies, Cairo University, El-Sheikh Zayed, 12588 Egypt

**Keywords:** Abu-Zaabal industry, Soil, Heavy metal contamination, Inductive coupled plasma optical emission spectrometry, Calibration-free picosecond laser-induced plasma spectroscopy (CF-Ps-LIPS), Environmental monitoring, Environmental impact, Natural hazards, Mineralogy, Environmental monitoring

## Abstract

Excessive fertilizer and chemical usage have led to soil contamination by toxic heavy metals near the Abu-Zaabal industrial complex in Egypt. We introduce a groundbreaking calibration-free methodology using ultrafast Picosecond Laser-Induced Plasma Spectroscopy (CF-Ps-LIPS) for quantifying contaminant elements (Cd, Zn, Fe, Ni) in soils near Egypt’s Abu-Zaabal industrial complex. This study pioneers applying 170 ps laser pulses (Nd: YAG, 1064 nm) to achieve calibration-free analysis, eliminating matrix-matched standards and offering ± 1% agreement with ICP-OES. By integrating plasma diagnostics (electron density N_e_ = 1.2–1.5 × 10^17^ cm^− 3^ and temperature T_e_ = 8508–10,275 K), we establish CF-Ps-LIPS as a rapid, minimally invasive tool for on-site environmental monitoring, validated through spatial contamination gradients linked to wind patterns. Concentrations of Cd (25.1–136.5 mg/kg), Zn (19.8–146.9 mg/kg), Fe (59.7–62 mg/kg), and Ni (119.4–157.8 mg/kg) were analyzed across seven sampling sites. The seventh site was used as a test sample of unknown concentration to validate CF-Ps-LIPS. Utilizing the Boltzmann distribution with plotting techniques enables precise plasma electron density and temperature determination under local thermodynamic equilibrium (LTE) conditions. The CF-Ps-LIPS study revealed significant concentration variations dependent on trace metal type, sampling location, and facility orientation. The CF-Ps-LIPS method provides calibration-free, rapid, and accurate detection of metal contaminants in Egyptian soils for the first time. This methodology significantly advances environmental monitoring and soil contamination analysis, allowing on-site assessments with higher efficiency and reliability.

## Introduction

Soil contamination by toxic elements (e.g., Cd, Ni) poses critical risks to ecosystems and human health, particularly near industrial zones like Egypt’s Abu-Zaabal complex. Elements in nature characterized by substantial atomic weights and a minimum density of 3 g/cm^3^ fall under the classification of heavy metals. Several metallic elements, including iron, zinc, copper, lead, chromium, cadmium, mercury, arsenic, and manganese, are indispensable for adequately functioning and maintaining biological systems in living organisms. However, when elevated concentrations of these elements are present, they can threaten human health and environmental integrity^1^. These contaminant elements have different sources, effects, and mechanisms of toxicity^2^. The heavy metals that emanate in substantial quantities from chemical manufacturing facilities represent the most hazardous contributors. Anthropogenic sources, such as industrial facilities, residential areas, vehicles, and the overuse of fertilizers and herbicides, have gained increasing importance as the human population grows^3,4^. The chemical transformations that heavy metals undergo in the soil can be affected by factors such as the type of soil, the presence of other chemicals, and the pH of the soil. These transformations can make the metals more or less mobile, bioavailable, and highly toxic^5^.

The two main techniques for analyzing heavy metals in soils and sediments are solution-based and direct analytical techniques^6,7^. Solution-based techniques encompass methodologies that employ liquid solvents and potent acids to extract, segregate, or quantify the attributes of matter contingent upon their interaction with electromagnetic radiation. Within this category, notable methodologies include inductively coupled plasma optical emission spectroscopy (ICP-OES), inductively coupled plasma mass spectrometry (ICP-MS), and atomic absorption spectrometry (AAS)^6^. Inductively Coupled Plasma Optical Emission Spectroscopy (ICP-OES) represents a method used in the current study for its merits. It operates by harnessing a high-energy plasma source in conjunction with an optical emission spectrometer, which collectively enables the quantitative determination of the elemental composition within a specimen. The rationale for selecting this technique is grounded in its remarkable attributes, including its exceptional sensitivity in detecting trace elements at concentrations as low as parts per billion (ppb).

Furthermore, it can analyze an extensive array of elements, from lithium to uranium, within a single sample. These features collectively underscore its unparalleled efficacy and versatility in elemental analysis. Crucially, the technique delivers outcomes marked by exceptional precision and accuracy, making it ideally suited for rigorous quantitative analysis of elemental concentrations that can be determined^8,9^. Operating ICP-OES instruments involves handling potentially hazardous chemicals and gases, necessitating strict adherence to safety protocols to mitigate risks to personnel and the environment^10^. The ICP-OES technique has disadvantages, including high cost, matrix effects, and sample preparation^8^. ICP-OES has been combined with picosecond laser-induced plasma spectroscopy (Ps-LIPS) for soil samples as a reference for the most advanced developed method to detect contaminant elements^11^.

Direct analytical methodologies, namely Laser-Induced Breakdown Spectroscopy (LIBS), Energy-Dispersive X-ray Spectroscopy (EDS), and X-ray Fluorescence Spectroscopy (XRFS), exhibit minimal or, in some instances, negligible prerequisites for sample preparation. Consequently, these techniques offer swifter and cost-effective analysis. XRFS excels in examining elements in substantial or moderate quantities. In contrast, LIBS and Laser Ablation-Inductively Coupled Plasma Mass Spectrometry (LA-ICP-MS) prove proficient in scrutinizing elements found in minute or exceedingly minute concentrations. Mitigation of interference and matrix effects can be attained through meticulous optimization of instrumental parameters and operational conditions^6^. The study used two analyzing contaminant elements techniques: LA-ICP-OES and LIBS^12,13^.

Laser-Induced Plasma Spectroscopy (LIPS) is a potent analytical technique for determining elemental composition within diverse materials. It accomplishes this by scrutinizing the emitted light from plasma generated via a laser pulse. The resulting plasma light offers distinct spectral lines that correspond to the elements present within the specimen. An exhaustive examination of this plasma light spectrum enables the precise identification of the sample’s elemental composition. Moreover, elemental concentrations can be accurately determined by analyzing the intensity of spectral lines, utilizing advanced techniques such as calibration or chemometric methods^14^.

LIPS boasts a multitude of distinctive advantages. It can analyze various material types, encompassing solids, liquids, gases, and aerosols. Additionally, LIPS can accommodate samples of varying geometries and sizes, including powders, pellets, wires, and thin films. Importantly, this technique excels in non-invasive analysis, permitting investigations to be conducted remotely and on-site, thus obviating the need for sample preparation or physical contact with the material. It is a minimally invasive technique, requiring only minute sample quantities, and offers rapid measurements, yielding results in a fraction of a second. LIPS further distinguishes itself through its capacity to simultaneously analyze multiple elements^15^. The versatility of LIPS finds application across diverse fields, spanning industrial, environmental, geological, archaeological, and biomedical studies^15^. This approach fundamentally hinges upon examining the laser ablation process^16,17^.

The analysis of contaminant elements utilizing Laser-Induced Plasma Spectroscopy (LIPS) has garnered substantial attention within scientific inquiry. Various studies and research endeavors have contributed to this burgeoning field. M. Hassan et al. employed LIPS as a minimally invasive method to quantify lead concentrations within soil and plant tissues^18^. In another context, Farooq et al. harnessed LIPS to evaluate soil samples collected from diverse desert regions in Riyadh City, Saudi Arabia^19^. Their study, conducted in the Haier basin near Riyadh, Saudi Arabia, focused on identifying elements such as chromium (Cr), manganese (Mn), copper (Cu), cadmium (Cd), magnesium (Mg), and iron (Fe) in the water samples. In a separate study, A. A. I. Khalil and O. A. Labib employed dual-pulsed LIPS to investigate the presence of micro-toxic elements in various coffee brands^20^. Their research focused on detecting elements such as aluminum (Al), lead (Pb), zinc (Zn), and chromium (Cr) in coffee samples.

Furthering the exploration of LIPS, Ahmed et al. monitored the toxicity associated with antidiabetic tablet drugs. This endeavor entailed a novel methodology that amalgamated calibration free-laser-induced plasma spectroscopy (CF-LIPS) and laser ablation-time-of-flight mass spectrometry (LA-TOF-MS)^21^. K. Elsayed et al. introduced an innovative protocol for quantifying phosphorus concentrations within phosphogypsum waste samples via calibration-free LIPS^22^. Lastly, A. Nassef and Y. Gamal showcased the utility of nanosecond laser-induced plasma spectroscopy (ns-LIPS) as a technique for characterizing contaminant elements within Nile River sediments^23^.

Traditional methods such as ICP-OES require extensive sample preparation and calibration, limiting their utility for rapid field analysis. While Laser-Induced Breakdown Spectroscopy (LIBS) offers direct analysis, conventional nanosecond lasers suffer from matrix effects and plasma instability. Recent advancements in ultrafast lasers (e.g., 170 ps pulses) enable precise calibration-free quantification by minimizing thermal ablation^24,25^. Building on this, we present the first application of CF-Ps-LIPS for soil analysis in Egypt, addressing spatial contamination patterns influenced by wind dynamics—a critical gap in prior LIBS studies^26^. In this study, we focus on making calibration-free curves from the concentration of the contaminant elements in the collected soil samples detected by a developed method composed of ICP-OES and Ps-LIPS together to utilize CF-Ps-LIPS method on the studied Egyptian soil samples for the first time. Plasma electron temperature and density are determined via the Boltzmann plot method and the Boltzmann equation. Accurate measurement and correction for these plasma parameters are essential for achieving precise and reliable quantitative calibration-free quantitative results.

## Methodology

### Samples collection and samples Preparation

#### Samples collection

**Fig. 1:**
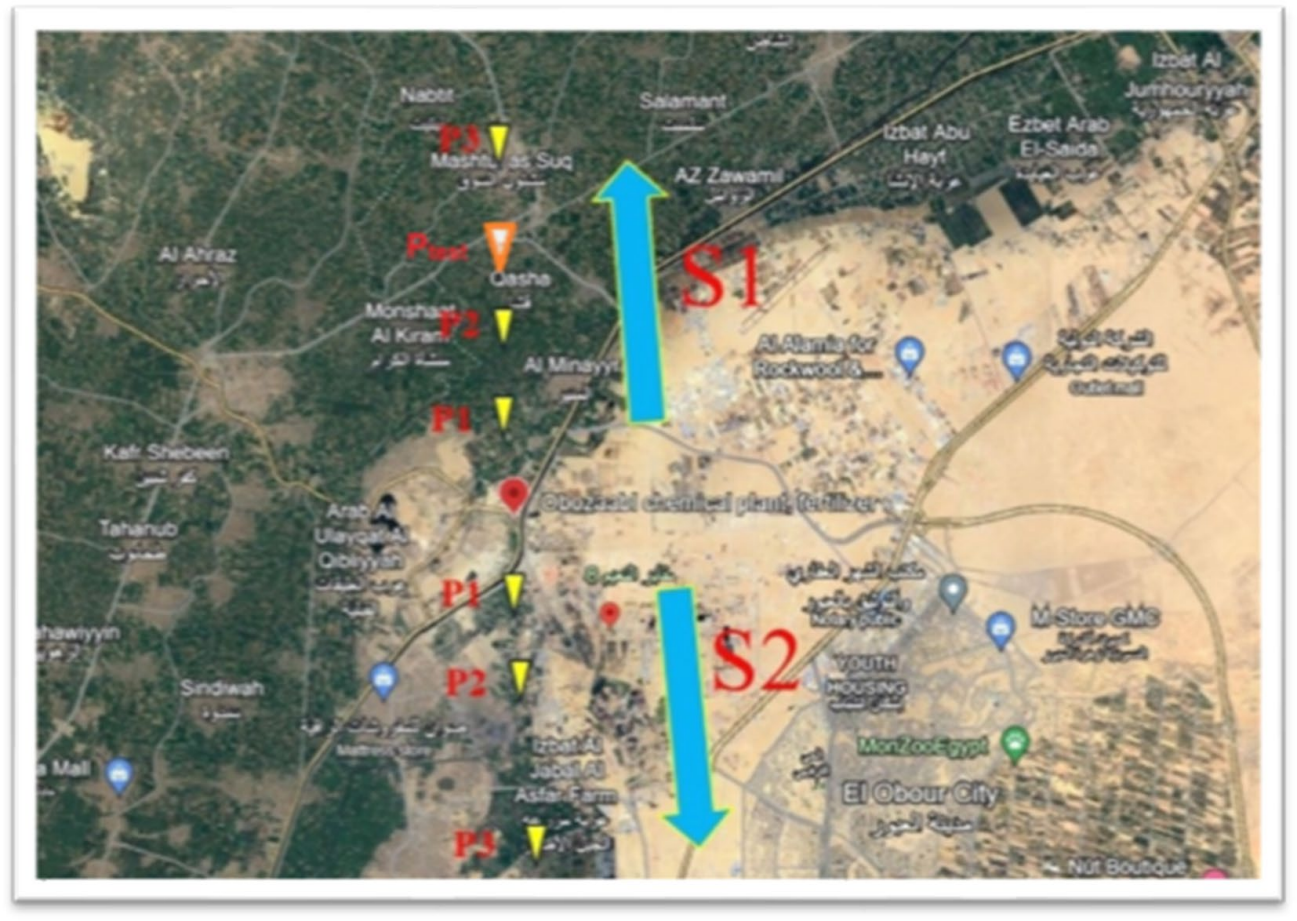
The studied site where the samples were collected in two directions (S1) and (S2). The map was generated under fair use for non-commercial scholarly purposes “Map data © [2023] Google, [https://www.google.com/maps] under free reuse CC BY license for for academic purposes (https://about.google/brand-resource-center/products-and-services/geo-guidelines/). Google Maps as a base layer [Year of Access is 6/2023]. The study site markers were subsequently added using Microsoft Paint((Microsoft Paint (v6.4) under Windows 10)).

In the winter of 2022, a soil sampling study was conducted in an agricultural area near Al-Khanka City, located west of the Nile Delta in Egypt. Six soil samples were collected from a region of approximately two km² surrounding the Abu-Zaabal industrial complex, which specializes in chemicals and fertilizer production. The locations of the collected soil samples with landmarks on the map are shown in Fig. 1. The soil sampling was conducted in two primary directions: North (S1) and South (S2). In each direction, three samples were collected at different locations (P1, P2, and P3) at 200, 750, and 2000 m from a reference point, respectively. This systematic approach ensured comprehensive coverage of the area.

Additionally, a test sample (P_test_) was collected from the North direction (S1) at a distance of 1000 m. This extra sample was explicitly designated to verify the concept of the CF-Ps-LIPS method and estimate concentrations using the established curve, adding a critical validation component to the study. A rigorous soil sampling protocol was implemented at each station to collect representative specimens while minimizing heterogeneity. The one-square-meter station area was divided into four equidistant sectors, with 1–2 kg samples extracted from a 10–20 cm depth at each vertex using manual excavation techniques. The four discrete samples were thoroughly homogenized through manual mixing to generate a composite sample that accurately characterizes the average pedological properties of the station, effectively integrating any fine-scale variability. Each composite specimen was securely stored in a resealable polyethylene bag to preserve sample integrity and prevent contamination. This approach ensures the collection of high-quality soil samples that reliably represent the conditions at each station, enabling robust analyses. By adhering to stringent sampling and preservation protocols, the study aims to obtain representative specimens that accurately reflect the pedological characteristics within the sampled area. Further specifics regarding the soil sampling, processing, and quality control procedures can be found in our previous work^27^.

#### Samples Preparation for ICP-OES analysis

The powdered soil samples were meticulously prepared for Inductively Coupled Plasma Optical Emission Spectroscopy (ICP-OES) analysis using the wet digestion method. An accurately measured sample of about one gram of the powdered soil was treated with a blend of concentrated nitric acid (HNO_3_) and hydrochloric acid (HCl) for digestion. After complete digestion, the solution was filtered through Whatman filter paper no. 41 into a calibrated 50 ml volumetric flask. The filtrate was then diluted to the mark with double-distilled water, achieving a final volume of 50 ml for subsequent elemental analysis. Standard solutions of 1000 µg/ml for four trace elements-(Cadmium (Cd), Nickel (Ni), Zinc (Zn), and Iron (Fe))-were acquired from Scharlau Chemie Co., Spain, and utilized for instrument calibration. The nitric acid and hydrochloric acid used were of analytical reagent (AR) grade, sourced from Merck Specialist Chemical Limited. For accurate volume measurements during the preparation and dilution processes, a calibrated micropipette ranging from 100 µl to 1000 µl was employed^28,29^.

#### Samples preparation for Ps-LIPS analysis

The Ps-LIPS technique requires a solid sample with a flat and smooth surface to generate a stable and reproducible laser-induced plasma^27^.

After collection, the soil samples were homogenized through manual mixing and grinding and desiccated at 80 °C to remove moisture. One gram of the resulting homogenized powder from each sample was pressed into circular pellets using a hydraulic press. Soil samples were homogenized, desiccated at 80 °C, and sieved to < 50 μm (mesh #300) to ensure particle uniformity. Pellets (1 cm diameter, 4 mm thick) were compacted under 5 metric tons for 15 min, minimizing porosity and ensuring plasma reproducibility^30^. This protocol aligns with Fayek et al. (2025)^24^who demonstrated sub-ppm detection limits in plant matrices using identical preparation methods.

The use of pelletized sample material serves multiple analytical purposes for Ps-LIPS. Firstly, a smooth, flat surface facilitates reproducible positioning and focusing of the probing laser pulse. Secondly, the high-pressure compaction enhances surface integrity, minimizing potential sample fragmentation or ablation during spectral acquisition. Most critically, the density and cohesiveness of the pellet minimize sample porosity and eliminate gaps between particles. This surface integrity is essential to generate a plasma plume that is genuinely representative of the bulk sample composition in an average sense rather than biased by surface particles and heterogeneity. Plasma plume interaction with the surrounding atmosphere is also minimized in this geometry^27^.

### Sample analysis techniques

#### Inductively coupled plasma optical emission spectroscopy (ICP-OES)

A radial view Spectro Ciros CCD ICP-OES (Spectro Analytical Instruments, Kleve, Germany) determined the metal and contaminant elements that validated our Ps-LIPS calibrated results. The operating parameters were meticulously optimized to ensure stable plasma conditions and optimal analytical performance. High-purity argon (99.99%) served as the plasma, auxiliary, and nebulizer gas, with flow rates established at 15.0 L/min, 1.50 L/min, and 0.56 L/min, respectively. The plasma generator’s radio frequency (RF) power was maintained at 1.35 kW, while the vertical observation height was fixed at 7 mm above the load coil to enhance analyte emission intensity. To assess plasma robustness, the intensity ratio of Mg II (280.2 nm) to Mg I (285.2 nm) was employed as a practical criterion, using a 20 mg/L magnesium solution. By varying the applied RF power from 1300 W to 1500 W, the Mg II/Mg I ratio increased from 6.00 to 8.39, indicating improved plasma stability. Under the optimized conditions, a Mg II/Mg I ratio of 7.94 was achieved, demonstrating a sufficiently robust plasma capable of minimizing potential matrix effects and ensuring consistent analytical performance^8,9^.

The ICP-OES system was equipped with an argon saturation assembly to minimize the impact of atmospheric gases on plasma stability and to enhance the overall analytical signal. Sample introduction was optimized to ensure consistent and representative delivery of the analyte to the plasma, with a sample uptake time of 30.0 s, a stabilization delay of 10 s, and a rinse time of 10 s between samples. The time between replicate analyses was set to 5 s to allow for sufficient washout and to maintain a high sample throughput. Before the commencement of the study, the ICP-OES instrument underwent a rigorous calibration procedure to ensure accurate and precise measurements across the entire wavelength range and concentration domain of interest. As a routine procedure, the calibration process involved using certified multi-element standards, covering the expected concentration range of the target analytes in the samples. Wavelength calibration was performed using a mercury argon lamp to ensure proper alignment and peak identification. In contrast, intensity calibration used matrix-matched standards to compensate for potential matrix effects. By carefully optimizing the operating parameters, evaluating plasma robustness, and conducting thorough calibration procedures, the radial view ICP-OES system was well-suited for accurately and precisely determining metals and contaminants elements in the studied samples, ensuring reliable and reproducible results throughout the investigation^8,9^.

#### Picosecond laser induced plasma spectroscopy (Ps-LIPS) setup

**Fig. 2:**
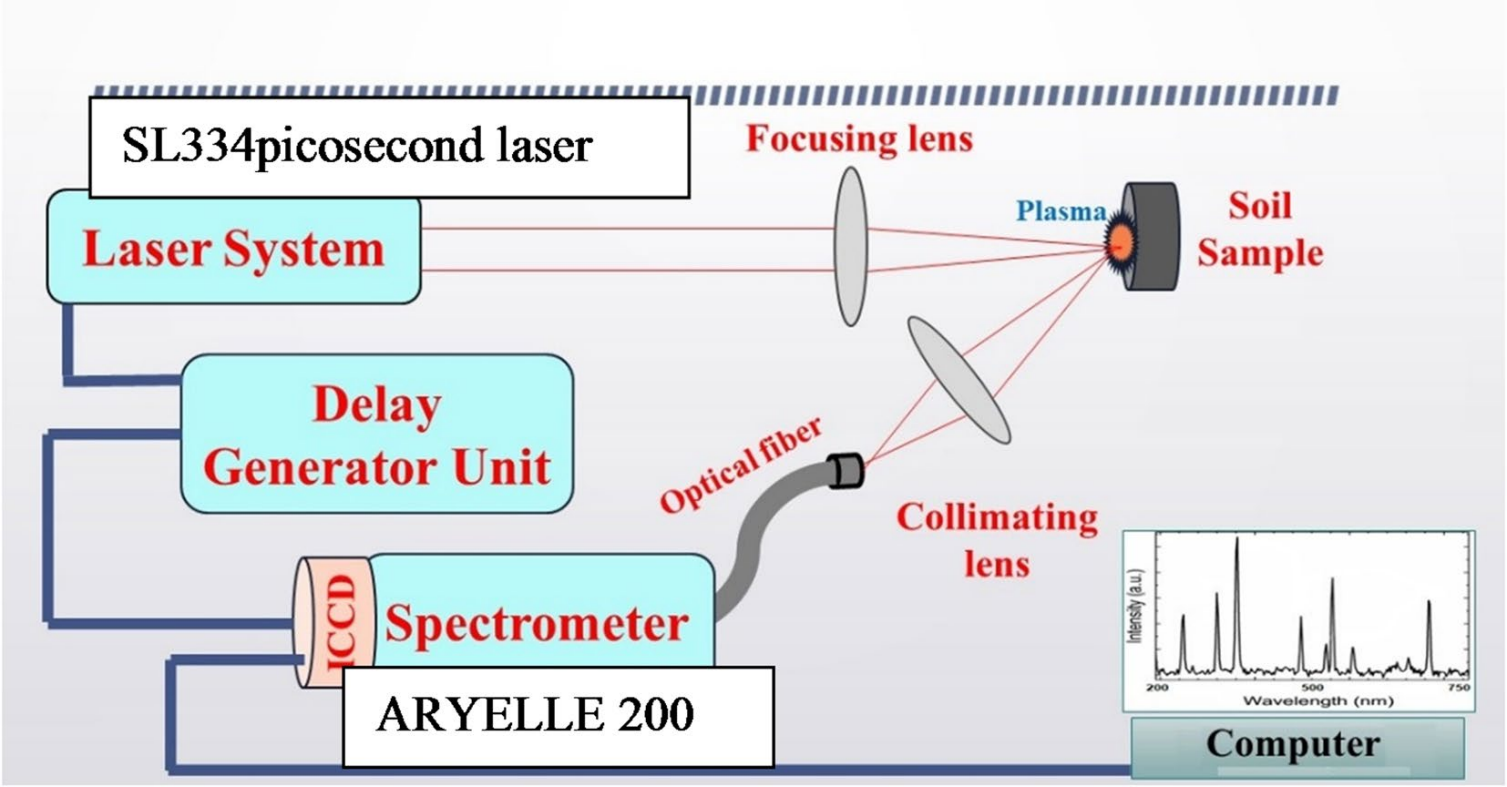
Schematic diagram of a high-accuracy laser-induced plasma spectroscopy.

Figure 2 represents the schematic diagram of a high-accuracy laser-induced plasma spectroscopy system setup, which depends on the ultrafast high-power picosecond laser and high-resolution picometer spectrometer. The Ps-LIPS (Plasma Spectroscopy with Laser-Induced Plasma Spectroscopy) setup utilized in this study has been previously detailed by our research group^31,32^. The experimental configuration features a high-power, ultrafast Nd: YAG Q-switched laser (Model SL334, Eksapla, Lithuania), which operates at a near-infrared wavelength of 1064 nm. This laser generates picosecond pulses with a duration of 170 ps and a repetition frequency of 5 Hz, delivering an energy output of 100 mJ per pulse with an energy tolerance of ± 4%. The laser pulses are focused onto the soil sample surface using a plano-convex quartz lens with a focal length of 150 mm, leading to a focused spot size of approximately 0.7 mm (± 0.1 mm). The energy of the laser pulses is accurately measured using a high-precision laser power meter (Model 11 Maestro, Standa LTD, Lithuania). Upon interaction with the soil, the laser induces the formation of a plasma plume, from which emitted light is collected by a precisely arranged optical system. This light is transmitted via an optical fiber positioned 7 ± 0.2 cm from the plasma origin at a 45-degree angle relative to the laser-plasma axis. The optical fiber directs the light to a sophisticated Echelle spectrometer (ARYELLE 200, Laser Technik Berlin GmbH, Germany), which operates in the picometer range. The ARYELLE 200 spectrometer, employed for PS-LIPS analysis, boasts a calibrated resolving power ranging from 75,000 to 150,000 across the detector’s operational bandwidth of 192 to 750 nm. This high resolving power enables the discrimination of two emission lines separated by as little as 0.001 nm at 500 nm. However, full resolution may not be achievable at longer wavelengths due to the inherent characteristics of the intensified charge-coupled device (ICCD) detector array. The proprietary data acquisition software compensates for variations in resolving power across the spectral bandwidth by integrating signals from multiple pixels for each resolvable wavelength bin. Although the theoretical resolution changes with wavelength, the reported data is conditioned to maintain a consistent resolving power of 75,000 throughout the analyzed spectral region. For each soil sample, a series of 50 LIPS spectra were collected at discrete positions on the sample, with each spectrum generated from the aggregation of 50 laser pulses, resulting in a cumulative total of 2,500 laser pulses applied to each sample. Accurate determination and calibration of the resolving power as a function of wavelength are critical for quantitative spectral interpretation. Insufficient corrections can lead to peak broadening, distortion, and erroneous wavelength shifts. The manufacturer’s calibration protocol employs sub-picometer atomic emission line sources to benchmark instrument response across the operational bandwidth. These stringent characterization and correction procedures ensure that the reported resolving power 75,000 effectively resolves all active plasma emission lines, enabling precise quantification and comparison of ps-LIPS spectra. The spectrometer operates in conjunction with an ICCD camera (Andor I-Star), which features adjustable amplification gain (up to ×1000), gate duration (2 µs), and delay times (1000 ns) for capturing and analyzing dispersed spectral data with high precision. An XYZ micrometer sample holder is employed to accurately position new target areas for each laser pulse to ensure consistent sample analysis and mitigate the effects of sample heterogeneity. Additionally, a quartz window protects the optical fiber head from contamination due to evaporated material during the ablation process.

## Result and discussion

### ICP-OES result

**Fig. 3:**
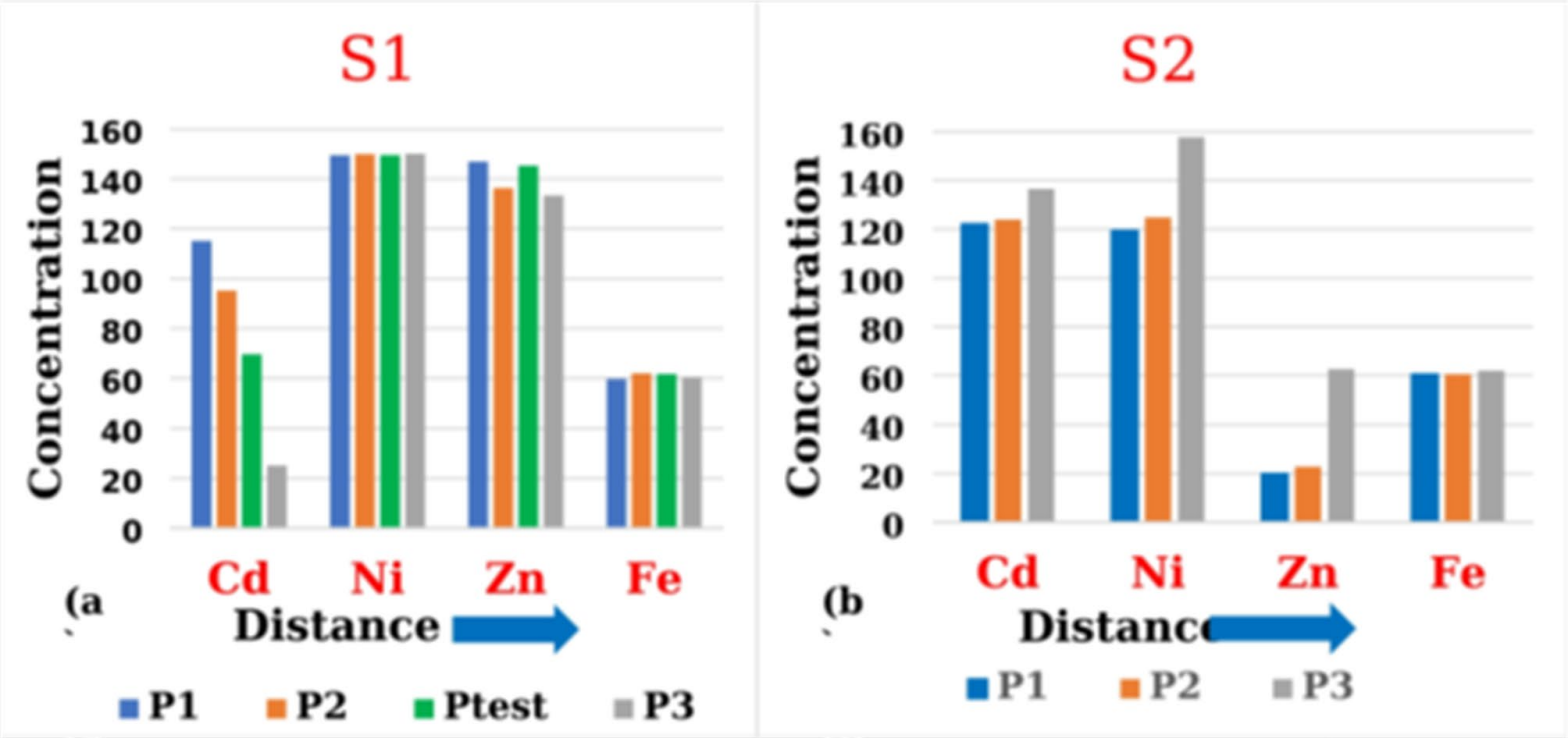
Concentrations of four elements for the two directions (**a**) (S1) and (**b**) (S2) according to the increase of the distance from the source of pollution.

Table 1; Fig. 3 present the concentrations of four elements—Cadmium (Cd), Nickel (Ni), Zinc (Zn), and Iron (Fe) in samples collected from sites S1 and S2. These sites form two transects from a central pollution source (Abu-Zaabal industrial complex), allowing for elemental distribution analysis relative to the contamination point. Sampling points P1, P2, and P3 are strategically positioned at increasing distances from this source, establishing a spatial gradient for observation and analysis. This experimental design allows for systematically evaluating the distribution and attenuation patterns of the targeted trace elements as a function of proximity to the pollution epicenter. By employing this approach, the study aims to elucidate the spatial extent and magnitude of contamination and potential trends in element partitioning and mobility within the investigated soil matrix. The data presented in tabular and graphical formats provide a wide-ranging overview of the spatial variability in trace element concentrations, enabling the identification of hotspots, gradients, and potential geochemical processes governing their distribution. These parameters are crucial for assessing environmental risks, developing targeted remediation strategies, and informing decision-making processes related to land use and soil management in the affected area. Table 1 is utilized to juxtapose the elemental concentrations within soil samples harvested from these locations, and the concentrations with error rates range from 0.5 to 1% and vary from one element to another. The table shows that the test sample P_test_ was determined to be compared with the estimated elemental concentrations using the CF-Ps-LIPS developed method. Figure 3a expounds on the elemental distribution along the S1 vector. It reveals a discernible diminution in Cd and Zn concentrations as the distance from the pollution epicenter increases. This pattern suggests a higher accumulation of these elements proximal to the source, with a tapering distribution moving away, likely attributed to the counteractive influence of prevailing wind currents at S1, which impedes the dispersion of pollutants. Conversely, the levels of Ni and Fe along S1 do not exhibit significant variation with distance, implying a more homogeneous distribution within the soil matrix or the potential influence of alternative sources.

**Table 1:** The ICP-OES measured the concentrations of contaminant elements found in the studied site in directions (S1) and (S2).

Elements concentration (mg/kg)	S1				S2		
	P1	P2	P3	*P*_test_	P1	P2	P3
Cd ± (0.98%)	115.10	95.15	25.10	69.62	122.10	123.95	136.50
Ni ± (0.63%)	149.50	149.90	150.05	149.53	119.40	124.85	157.80
Zn ± (0.77%)	146.90	136.35	133.30	145.33	19.80	22.78	62.60
Fe ± (0.51%)	59.70	61.95	60.50	61.67	60.50	60.55	62.00

Figure 3b addresses the distribution along the S2 vector, displaying a contrary trend. Here, the concentrations of Cd, Ni, Fe, and Zn manifest an elevation with increasing remoteness from the source. This trend suggests an initial dilution of these elements proximal to the source, followed by a progressive enrichment in the soil substrate at extended ranges. Such a distribution is likely facilitated by the alignment of S2 with the regional wind patterns, promoting the aerial transmission of pollutant particulates and enhancing soil deposition at greater distances. This phenomenon aligns with extant literature examining the wind-mediated translocation of particulate-bound contaminant elements. The vector and velocity of wind, in concert with the physicochemical characteristics of the particulates, such as size, density, and morphology, critically modulate the dispersion and ultimate deposition of heavy metal contaminants. The resultant distribution can extend the impact of pollution from point sources, such as mines, to distal ecosystems, engendering widespread environmental implications, including the contamination of air, hydrosphere, and biosphere^33,34^.

### Ps-LIPS results

#### Ps-LIPS spectrum studies

**Fig. 4:**
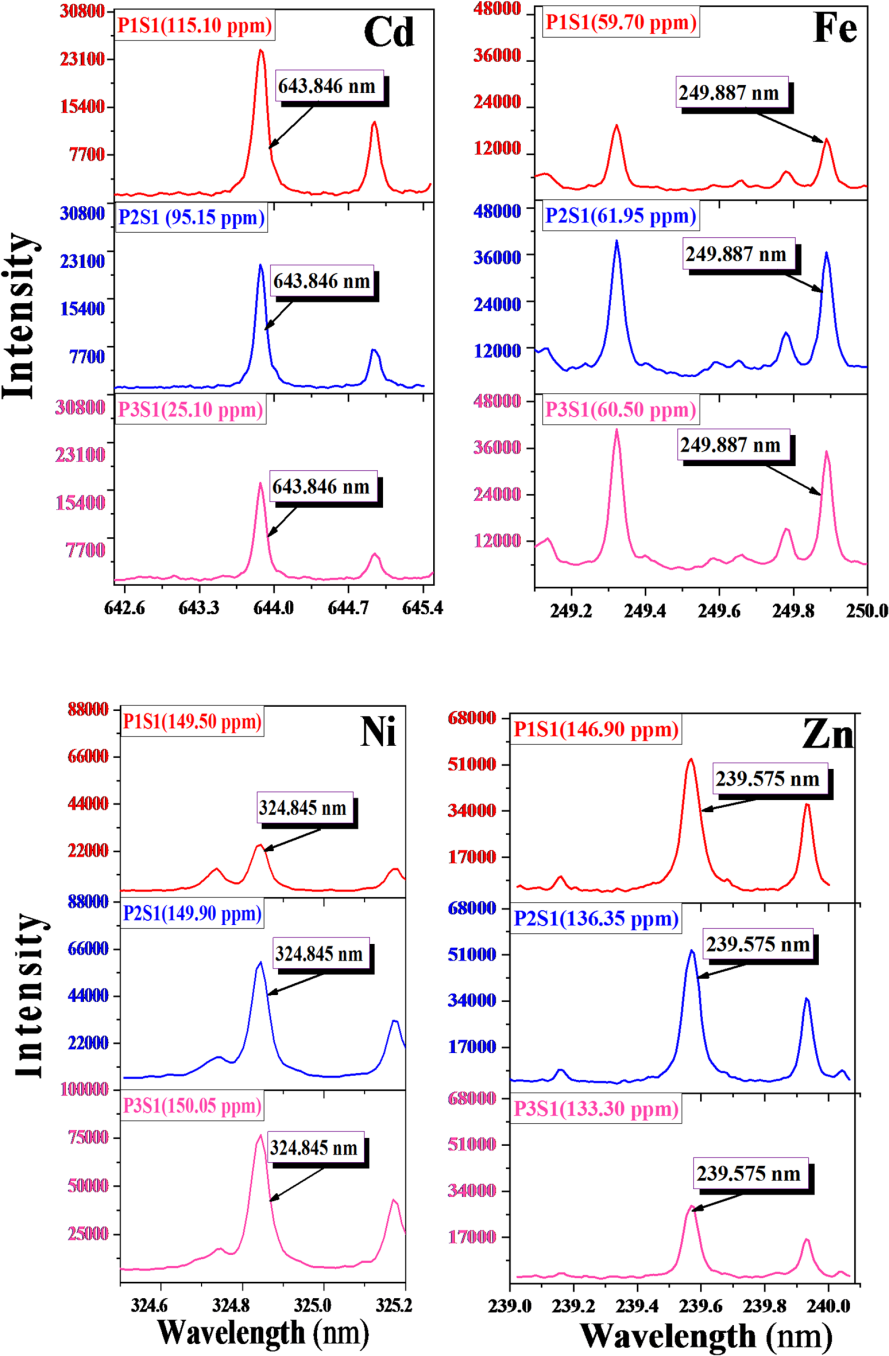
Illustrate high-resolution Ps-LIPS emission spectra of the wavelength of the four elements (Cd, Ni, Zn, and Fe) in the first direction (S1).

**Fig. 5:**
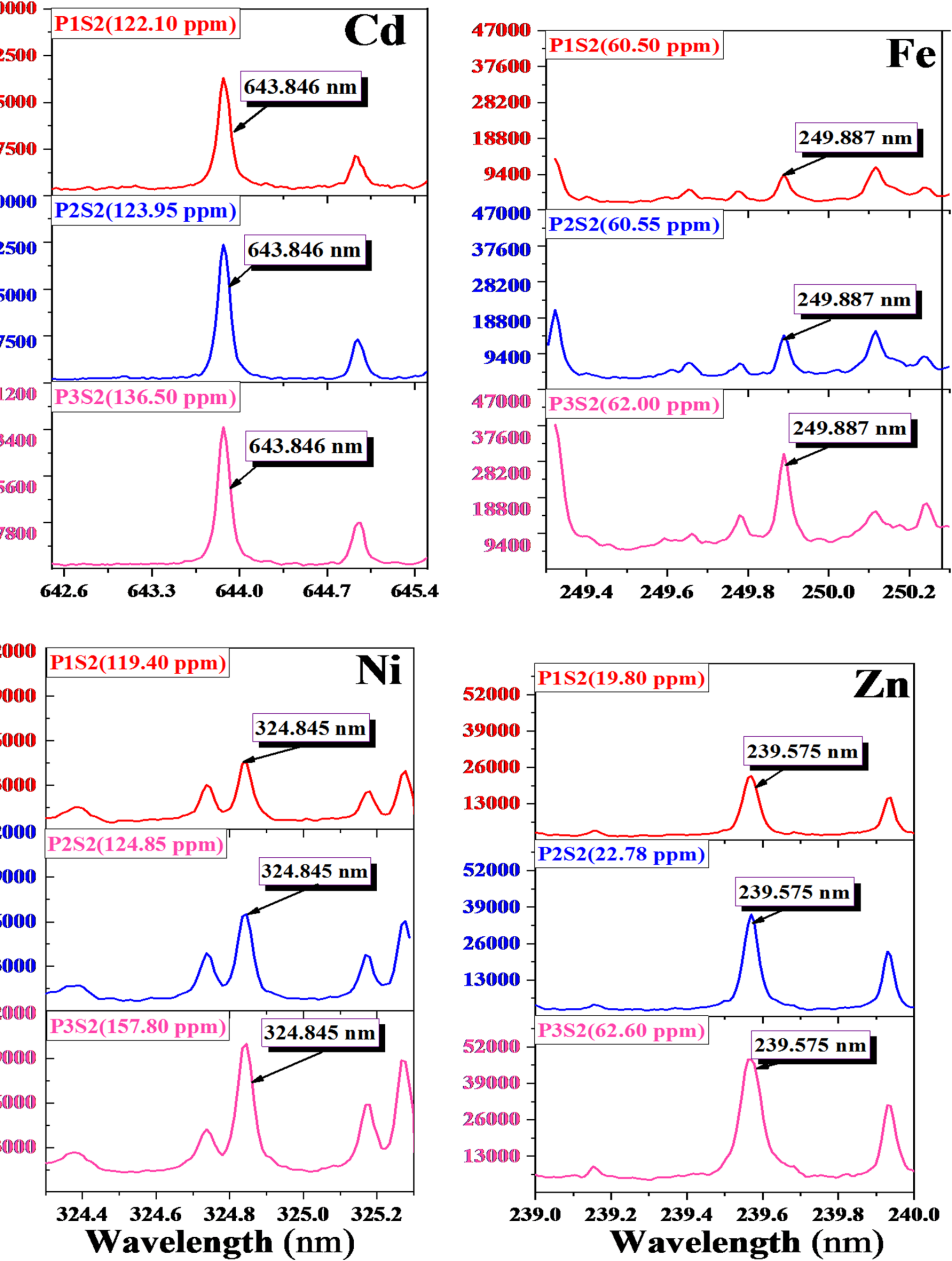
Illustrate high-resolution Ps-LIPS emission spectra of the wavelength of the four elements (Cd, Ni, Zn, and Fe) in the second direction (S2).

The qualitative analysis of the collected soil samples was performed using the Picosecond Laser-Induced Plasma Spectroscopy (Ps-LIPS) technique. Optical spectra were generated using a single shot from a near-infrared (NIR) picosecond Nd: YAG pulsed laser with a 170 ps pulse duration, 100 mJ pulse energy, 2 µs detection gate time, and 1000 ns delay. Figures 4 and 5 present high-resolution Ps-LIPS emission spectra for elements Cd, Fe, Ni, and Zn collected from three points (P1, P2, and P3) in two different sides concerning Abu-Zaabal industrial complex (Fig. 4 represents the north side in the direction S1 and Fig. 5 represents the south side in the direction S2). The selected spectral lines for these singly ionized elements are 643.847 nm (Cd), 249.887 nm (Fe), 324.846 nm (Ni), and 239.575 nm (Zn)^35^. Table 2 presents the spectral parameters, including experimental wavelength (λexp), corresponding theoretical lines from the NIST database, wavelength error (±Δλ), and transition configuration for each wavelength. All spectral data were obtained from the NIST Atomic Spectra Database^35^.

**Table 2:** The spectroscopic parameters of the contaminant elements ' spectral lines^35^.

Heavy metals	Selected wavelengths			
	λ_th_ (nm)	λ_exp_ (nm)	± ∆λ (pm)	Transition configuration
Cd	643.846	643.870	24	4d^10^5s4p → 4d^10^5s5d
Ni	324.846	324.845	1	3d^9^(^2^D)4s → 3d^8^(^3^f)4s4p(^3^P°)
Zn	239.575	239.569	6	4d^10^4s4p → 4d^10^5s8g
Fe	249.887	249.888	1	3d^6^4s^2^ → 3d^6^(^3^F2)4s4p(^3^P°)

**Fig. 6:**
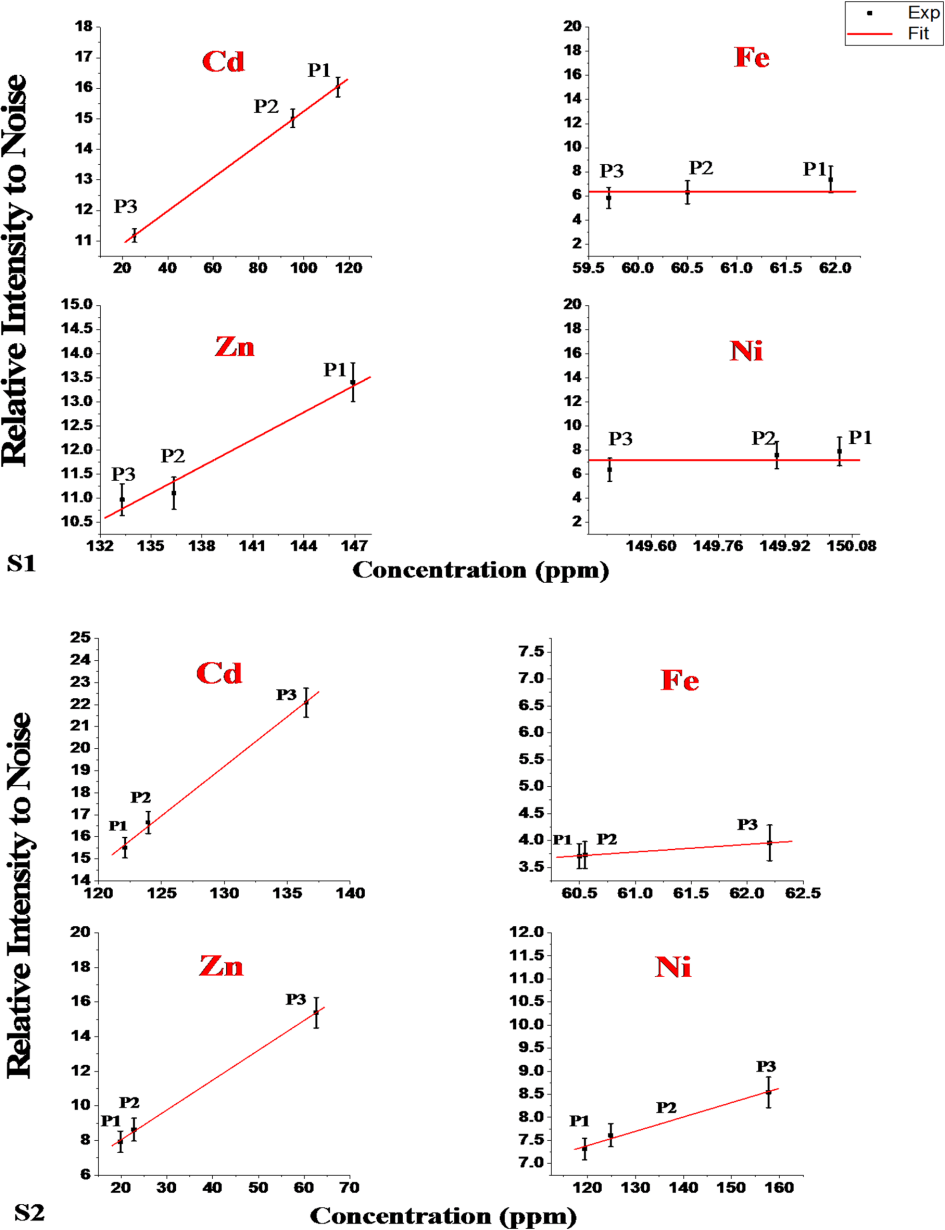
The correlation curves between the concentrations measured by ICP-OES and the Ps-LIPS signal intensity to noise for the selected spectral lines of soil samples for the two directions (S1) and (S2).

Figure 6 demonstrates the correlation between the relative intensity of the Ps-LIPS signal (compared to background noise) and the concentrations of selected elemental spectral lines in soil samples, as quantified by ICP-OES. In sample S1, a notable decrease in the relative intensity of spectral lines for cadmium and zinc is observed from P1 to P3, while the signal for iron and nickel remains relatively constant. Conversely, sample S2 exhibits a clear ascending trend in the relative intensity of spectral lines for all four trace elements (Cd, Zn, Fe, and Ni) from P1 to P3. This pattern suggests that the distance from an industrial facility influences the relative signal intensity, as evidenced in S1 and S2. The primary factor driving this variation is the increase in the mass ablation rate, which directly correlates with the elevated concentration of these elements in the samples. As the distance from the pollution source decreases, the quantity of material vaporized by the laser increases, resulting in stronger signal intensities for the elements of interest. This relationship between distance, elemental concentration, and signal intensity provides valuable insights into the spatial distribution of pollutants around the industrial site^36^.

### Plasma diagnostics

Plasma diagnostics within the (Ps-LIPS) technique entails measuring and analyzing laser-generated plasma’s physical and chemical properties, including its temperature, density, composition, and dynamic behavior. Through these diagnostics, valuable insights can be gained regarding the interaction between laser energy and matter. This information is pivotal in augmenting the analytical capabilities of Ps-LIPS, as well as in the advancement of calibration-free quantitative analysis methodologies^16,17,37^. Table 3 shows the spectroscopic parameters of the calcium atomic (Ca I) spectral lines used in plasma diagnostics obtained from NIST^35^.

**Table 3:** Demonstrates the Ca I spectral lines used in the plasma diagnostic^35^.

λ_(nm)_	A_ki ×10_^7^_(s_^−1^_)_	E_i(eV)_	Ek_(eV)_	g_k_	Lower level configuration	Upper level configuration
646.257	4.7	2.522	4.440	7	3p^6^3d4s ^3^D 2	3p^6^3d4p ^3^F° 3
428.936	6	1.879	4.769	3	3p^6^4s4p ^3^P° 0	3p^6^4p2 ^3^P 1
442.544	4.98	1.879	4.680	3	3p^6^4s4p ^3^P° 0	3p^6^4s4d ^3^D 1
527.027	5	2.525	4.877	5	3p^6^3d4s ^3^D 3	3p^6^3d4p ^3^P° 2
585.745	6.60	2.932	5.048	5	3p^6^4s4p ^1^P° 1	3p^6^4p2 ^1^D 2
612.222	2.87	1.885	3.910	3	3p^6^4s4p ^3^P° 1	3p^6^4s5s ^3^S 1

#### Plasma electron density

Electron density quantifies the concentration of electrons within a specified volume or region of a system. When a system is in Local Thermodynamic Equilibrium (LTE), the electron density can be accurately deduced using the Boltzmann distribution. This fundamental statistical distribution connects the system’s energy states and temperature to the likelihood of electrons occupying those states.

In the context of LTE, the Boltzmann distribution (denoted as Eq. 1) is instrumental in evaluating the electron density in various states of matter, including plasmas, gases, and solids. By applying this distribution, one can compute how the electron density varies across different locations within the system or at various energy potentials. This is particularly useful for understanding the electron distribution in systems where the electrons are in thermal equilibrium with their surroundings, such as in studying stellar atmospheres, semiconductor physics, and plasma diagnostics^11,38,39^.

1 $$\:{N}_{e}\approx\:\:\frac{{\varDelta\:\lambda\:}_{FWHM}\:\:}{2{W}_{s}}\times\:{10}^{16}.$$

Where N_e_ represents the electron density (in cm^− 3^), Δλ_FWHM_ represents the fundamental line width at half maximum, and W_s_ represents the electron Stark-broadening value of the element spectral line.

The Stark-broadening profile of the Ca I line at 646.257 nm is utilized as a reference standard for assessing electron density due to its relatively isolated spectral position, narrow linewidth, and absence of self-absorption effects^40^. According to H. R. Griem’s seminal work (1964), the mean Stark width (Ws) for the Ca I line at 646.257 nm is determined to be 0.0381 nm, corresponding to a plasma electron temperature of 10,000 K^41^.

**Fig. 7:**
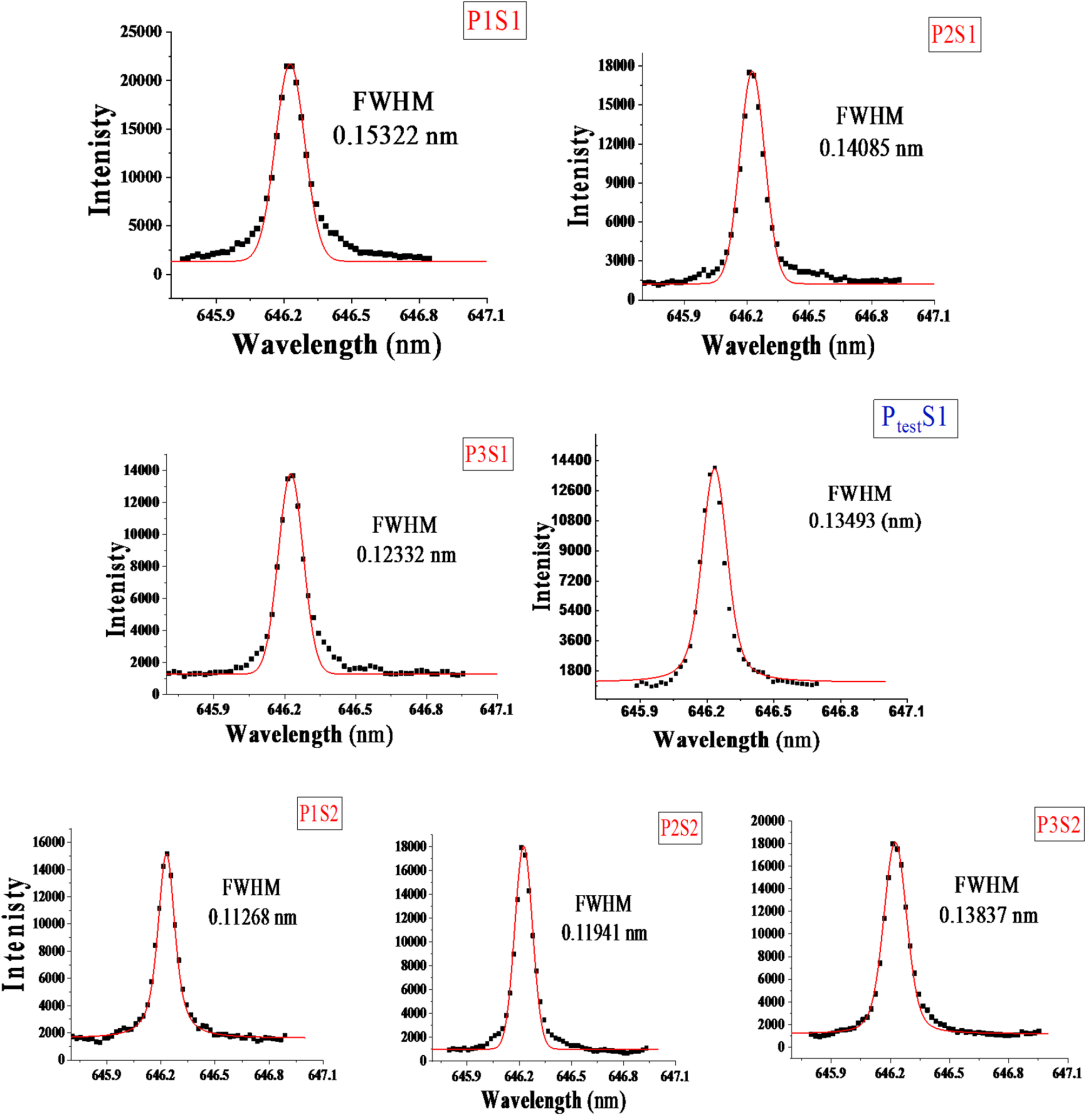
Voigt Line profile of Ca I line 646.257 nm of soil samples for the two directions (S1) and (S2).

The Origin professional version 2018 was used to fit the Ca I line 646.257 nm profile using the Voigt function to obtain the line full-width-half-max (Δλ_FWHM_), as shown in Fig. 7. We used the Voigt function because Voigt fitting is a convolution of both the Gaussian and Lorentzian profiles, combining their characteristics. Gaussian line profiles primarily result from instrumental broadening mechanisms such as spectrometer resolution. Meanwhile, Lorentzian line shapes are associated with natural broadening through interactions between the emitting particles and the surrounding plasma environment. In laser-induced plasma spectroscopy, the measured spectral profiles exhibit combined natural and instrumental broadening effects^42^.

The Voigt profile accounts for Gaussian instrument response and Lorentzian plasma interactions within a single function. Consequently, Voigt fitting often provides a much more accurate representation of experimentally observed line shapes than either independent function. This fitting avoids erroneous overfitting that may occur from applying two functions sequentially. The integrated area under the Voigt profile also enables more reliable spectral quantification and lower detection limits than Gaussian or Lorentzian fitting approximations.

The spectral line FWHM of the Ca 646.257 nm decreases from 0.15322 to 0.12332 nm ± 2.3% in order from P1 to P3 in S1. Nerveless, the spectral line FWHM of the Ca 646.257 nm increased from 0.11268 to 0.13837 nm ± 2.1% from P1 to P3 in S2. The variation of the mass ablation rate due to variation of the concentration of the elements matrix in the samples with increasing the distance, which depends on the wind direction in the studying site, considers the mean reason for the variation of the N_e_ with the sites and positions^34^.

The characteristics of laser-induced plasma depend on the elemental makeup of the sample matrix under examination. Soils typically contain major matrix elements, chiefly silicon, magnesium, and calcium, and minor elements, such as nickel, zinc, iron, and cadmium, often found in far lesser quantities. Significant variations in major element concentrations can fundamentally alter the matrix properties. However, changes in trace elements can also influence plasma parameters, even without altering the matrix identity, as previously demonstrated^43^. Kumar and colleagues evaluated calibration-free laser-induced plasma spectroscopy (CF-LIPS) and multivariate analysis methods for quantitative trace elemental analysis in their work. They examined certified soil reference materials spiked with supplemental trace elements at varying concentrations. Through monitoring diagnostic atomic emission lines, CF-LIPS revealed detectable changes in plasma electron temperature and number density correlated with increasing trace elemental spikes. Meanwhile, partial least squares regression analysis enabled robust trace elemental quantification across all concentrations investigated^43^.

**Fig. 8:**
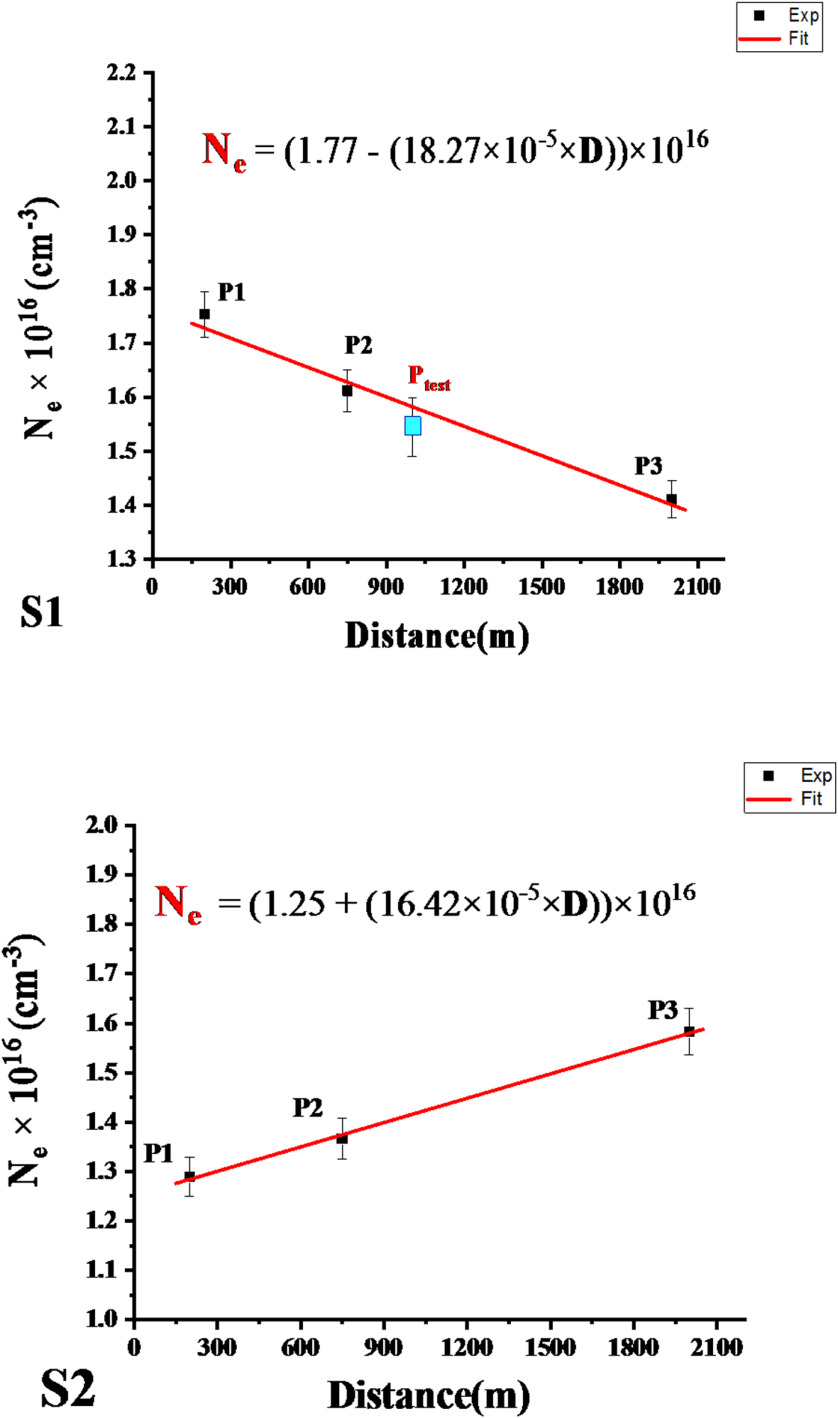
Calibration-free curves of electron density (N_e_) and Distance (D) for the two directions (S1) and (S2).

**Fig. 9:**
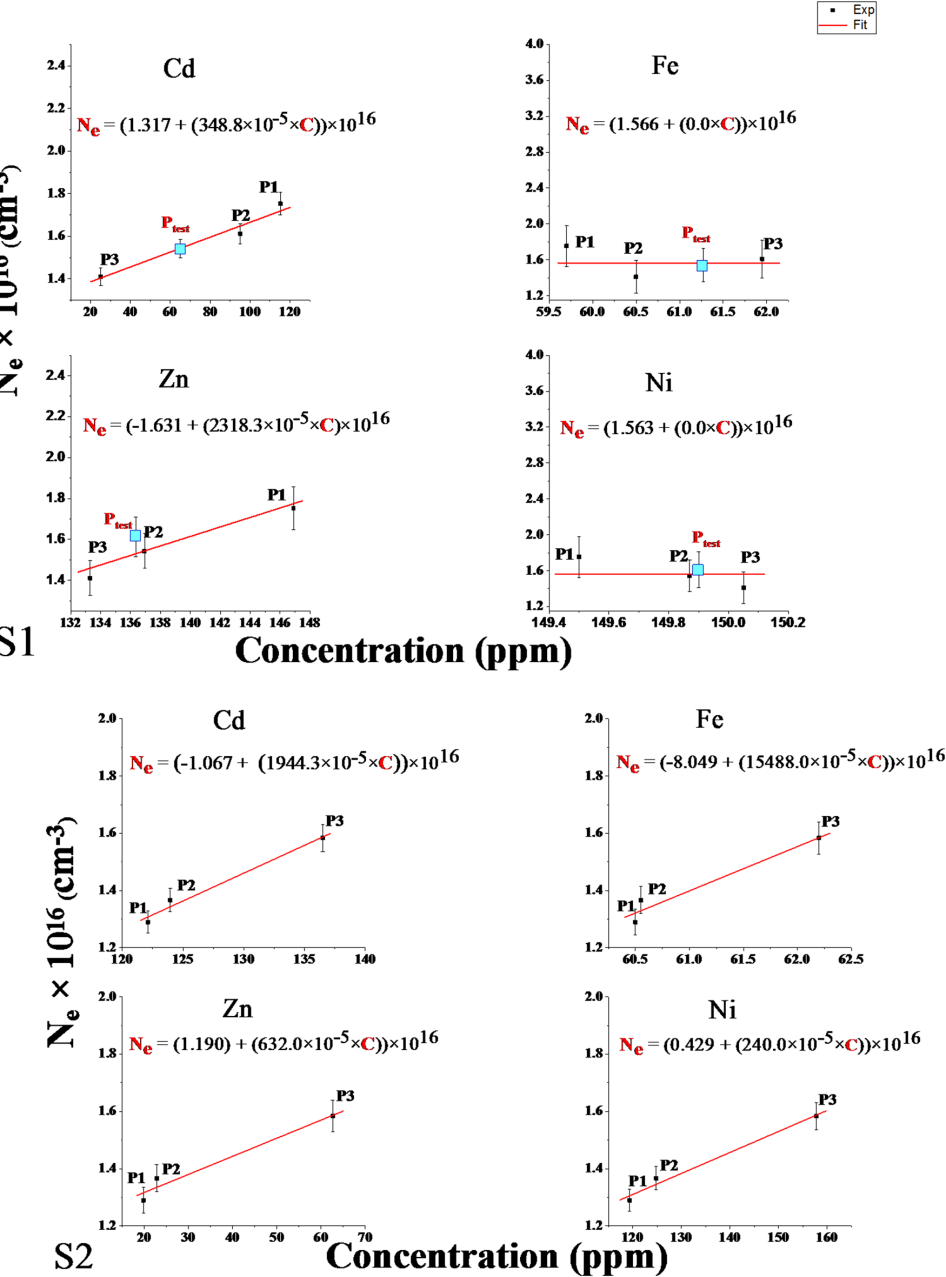
Calibration-free curves of electron density (N_e_) and concentrations (C) of contaminant elements for the two directions (S1) and (S2).

These analytical approaches highlight the sensitivity of laser-induced plasma to subtle trace elemental fluctuations without requiring extensive baseline data collection or matrix-matched calibration standards. As such, this study’s laser-induced plasma spectroscopy technique possesses the requisite sensitivity to both matrix-significant elements and trace constituents to characterize complex soil specimens adequately. Quantitative variations in the plasma emission spectra can thus be correlated to changes in trace elemental compositions, enabling discrimination between the studied samples. Figure 8 displays calibration-free linear regression curves that map the relationship between electron density and spatial distance for various sample locations along two opposing directions. This graphical representation offers a detailed examination of the electron density’s variation with the samples’ locations, highlighting the distribution and directional trends of the data. The observation and analysis of Fig. 8 show that N_e_ varied from 1.75 × 10^16^ to 1.41 × 10^16^ ± 2.4% and 1.2 × 10^16^ to 1.5 × 10^16^ ± 1.6% for S1 and S2, respectively. These trends verify that the dependence of the electron density on the distance and site direction from the source of pollution is related to variations in the sample mass ablation rate and concentration of elements matrix in the sample, as shown in Fig. 9.

The experimental formulas from Figs. 8 and 9 determined the electron density gradient with distance, as shown in Eqs. 3 and 4. The element concentrations in the sample matrix for both directions, S1 (opposite to wind direction) and S2 (with wind direction), are presented in Table 4. These results demonstrate that wind direction relative to the pollutant source affects the spatial distribution of heavy metal contamination by the following relationship (Eq. 2):

2 $$\:{\mathbf{N}}_{\mathbf{e}}\:\left({\mathbf{c}\mathbf{m}}^{-3}\right)=\text{a}\:+\:\left(\text{b}\times\:\text{D}\left(\text{m}\right)\right)\times\:{10}^{16}.$$

where N_e_ is the electron density, D is the distance from the fertilizer facility, and (a and b) are empirically derived constants.

3 $$\:{{\mathbf{N}}_{\mathbf{e}}\:\left({\mathbf{c}\mathbf{m}}^{-3}\right)}_{Opposite\:\:wind}=\left(1.77\right)-\left(18.27\:\times\:{10}^{-5}\times\:\:\text{D}\left(\text{m}\right)\right)\times\:{10}^{16}$$

4 $$\:{{\mathbf{N}}_{\mathbf{e}}\:\left({\mathbf{c}\mathbf{m}}^{-3}\right)}_{With\:\:wind}=\left(\:1.25\right)\:+\:\left(\:16.42\:\times\:{10}^{-5}\times\:\:\text{D}\left(\text{m}\right)\right)\times\:{10}^{16}.$$

The wind directionality dependence of heavy metal contamination established here highlights the utility of careful spatial sampling and rigorous data analysis in characterizing the environmental transport dynamics of airborne pollutants from point sources.

**Table 4:** Experimental empirical formula for calculating plasma electron density based on the concentrations of the contaminant elements.

Element	*N*_e_ (cm^−3^), C (ppm)	
	S1	S2
Cd	N_e_ = (1.317 + (348.8 × 10^−5^ × C)) × 10^16^	N_e_ = (− 1.067 + (1944.3 × 10^−5^ × C)) × 10^16^
Fe	N_e_ = (1.566 + (0.0 × C)) × 10^16^	N_e_ = (− 8.048 + (15488.0 × 10^−5^ × C)) × 10^16^
Zn	N_e_ = (− 1.631 + (2318.3 × 10^−5^ × C)) × 10^16^	N_e_ = (1.190 + (632.0 × 10^−5^ × C)) × 10^16^
Ni	N_e_ = (1.563 + (0.0 × C)) × 10^16^	N_e_ = (0.429 + (240.0 × 10^−5^ × C)) × 10^16^

#### Plasma electron temperature

The plasma temperature is obtained by plotting the logarithm of the intensity ratio of each line to a reference line versus the energy difference between their upper levels and finding the slope of the linear fit Using the Boltzmann plot method shown in Eq. 5^45^.

5 $$\:\text{ln}\frac{I\lambda\:}{{A}_{ki}{g}_{k}}=-\frac{1}{K{T}_{e}}{E}_{k}+\text{ln}\frac{FC}{U\left(T\right)}.$$

Here, I is the intensity of the spectral line, λ is the wavelength of the spectral line, K is the Boltzmann constant, U(T) is the partition function, A_ki_ is the transition probability, g_k_ is the statistical weight for the upper level, E_k_ is the excited-level energy, T_e_ is the electron temperature, F is an experimental factor, and C is the concentration of species.

**Fig. 10:**
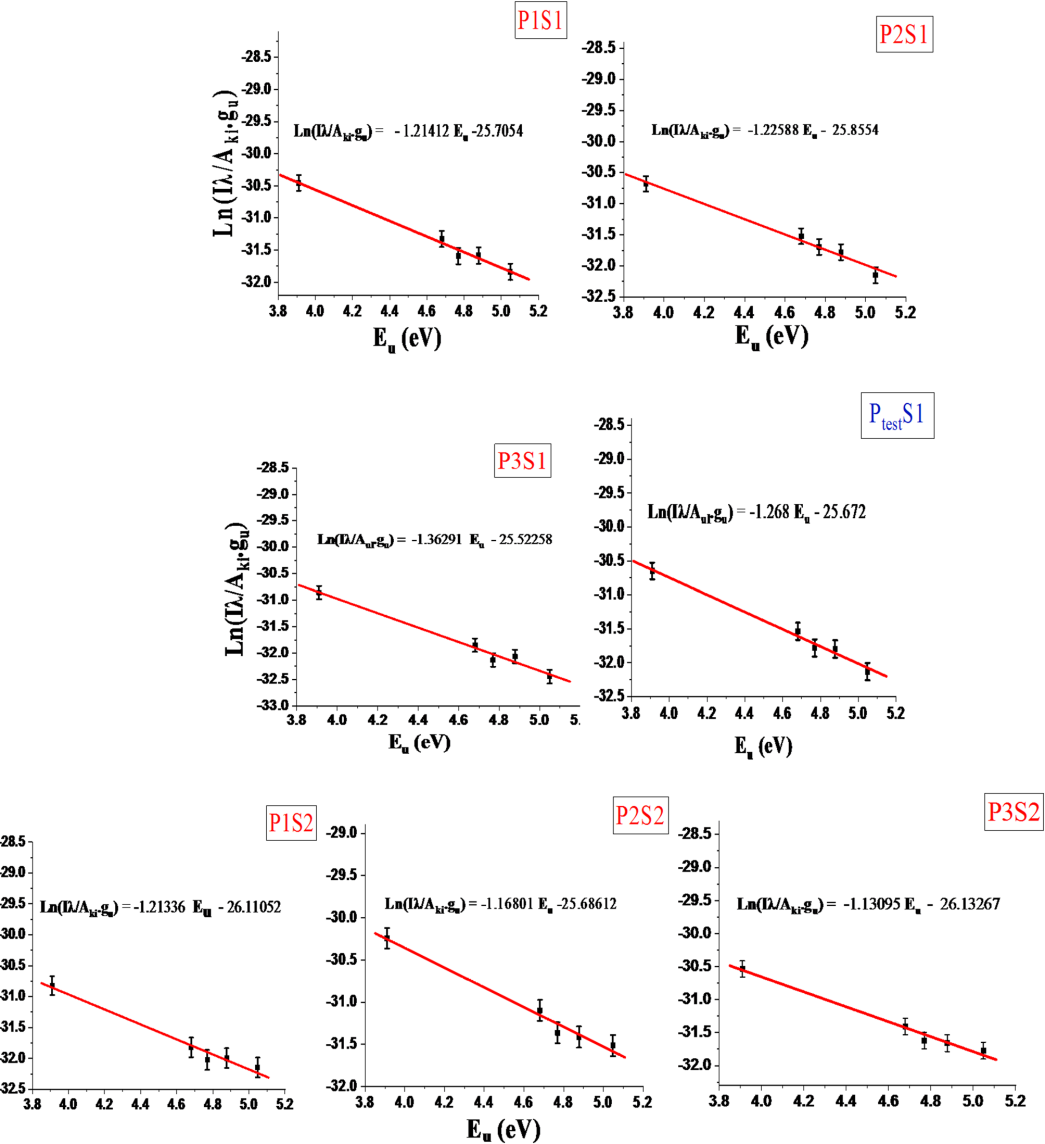
The Boltzmann plots using Ca I wavelengths (Table 3) for the two directions (S1) and (S2).

The Boltzmann plot method is a spectroscopic technique that uses the emission spectra of atoms or ions in a plasma to determine their temperature. It is based on the assumption that the plasma is in the condition of local thermodynamic equilibrium (LTE), which means that the population of the excited states follows the Boltzmann distribution^45,46^. The Boltzmann plot method requires measuring the intensity of several spectral lines from the same element with different excitation energies. Then, one plots the natural logarithm of the intensity ratio of each line to a reference line versus the energy difference between their upper levels and the reference line. The slope of the linear fit gives the inverse of the temperature (k), where k is the Boltzmann constant, and T is the temperature^46,47^. The reference lines were chosen in our study for Ca I with wavelengths (428.936, 442.544, 527.027, 585.745, and 612.222 nm) these lines were selected because it has a high intensity and a low self-absorption coefficient. Figure 10 illustrates the slope of the Boltzmann plots where the ln (I.λ/A_ki_.g_u_) is considered for each exciting upper energy level E_u_.

**Fig. 11:**
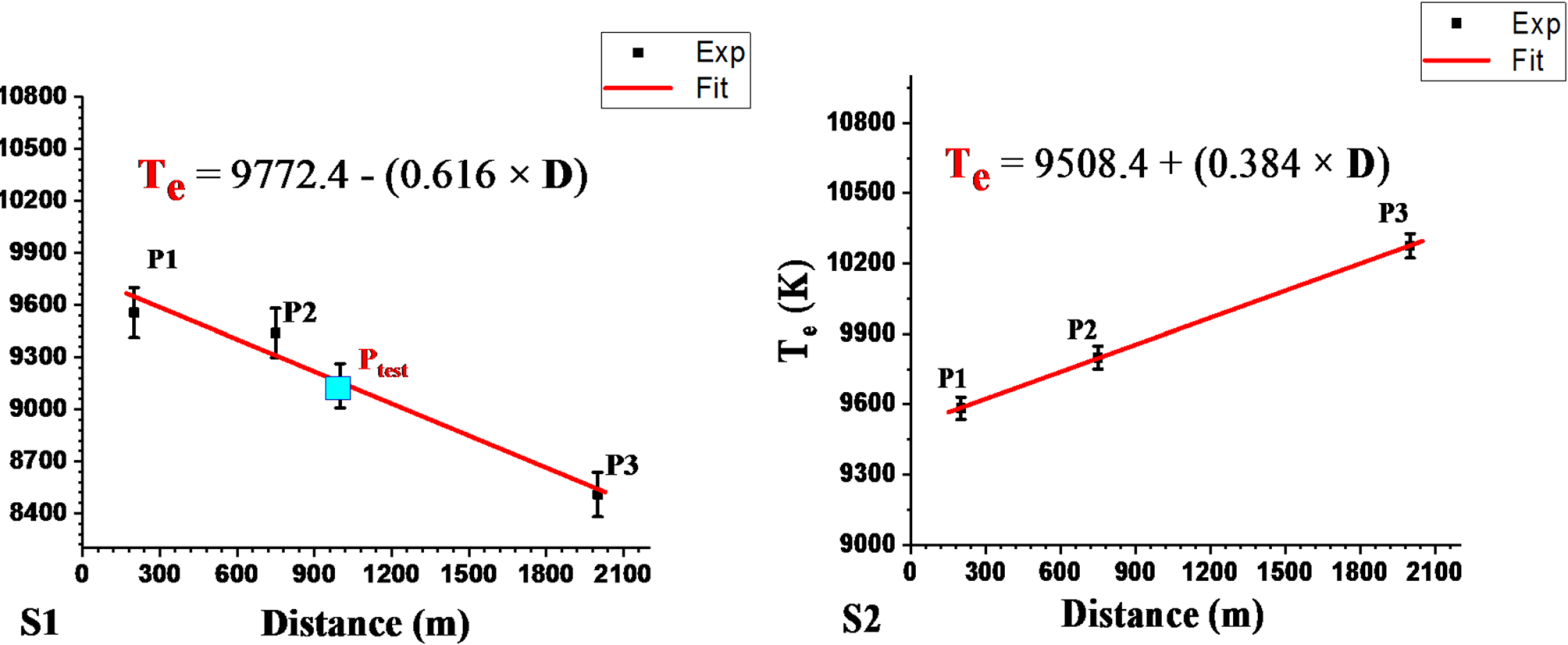
Calibration-free curves of Electron Temperature (T_e_) and Distance (D) for the two directions (S1) and (S2).

**Fig. 12:**
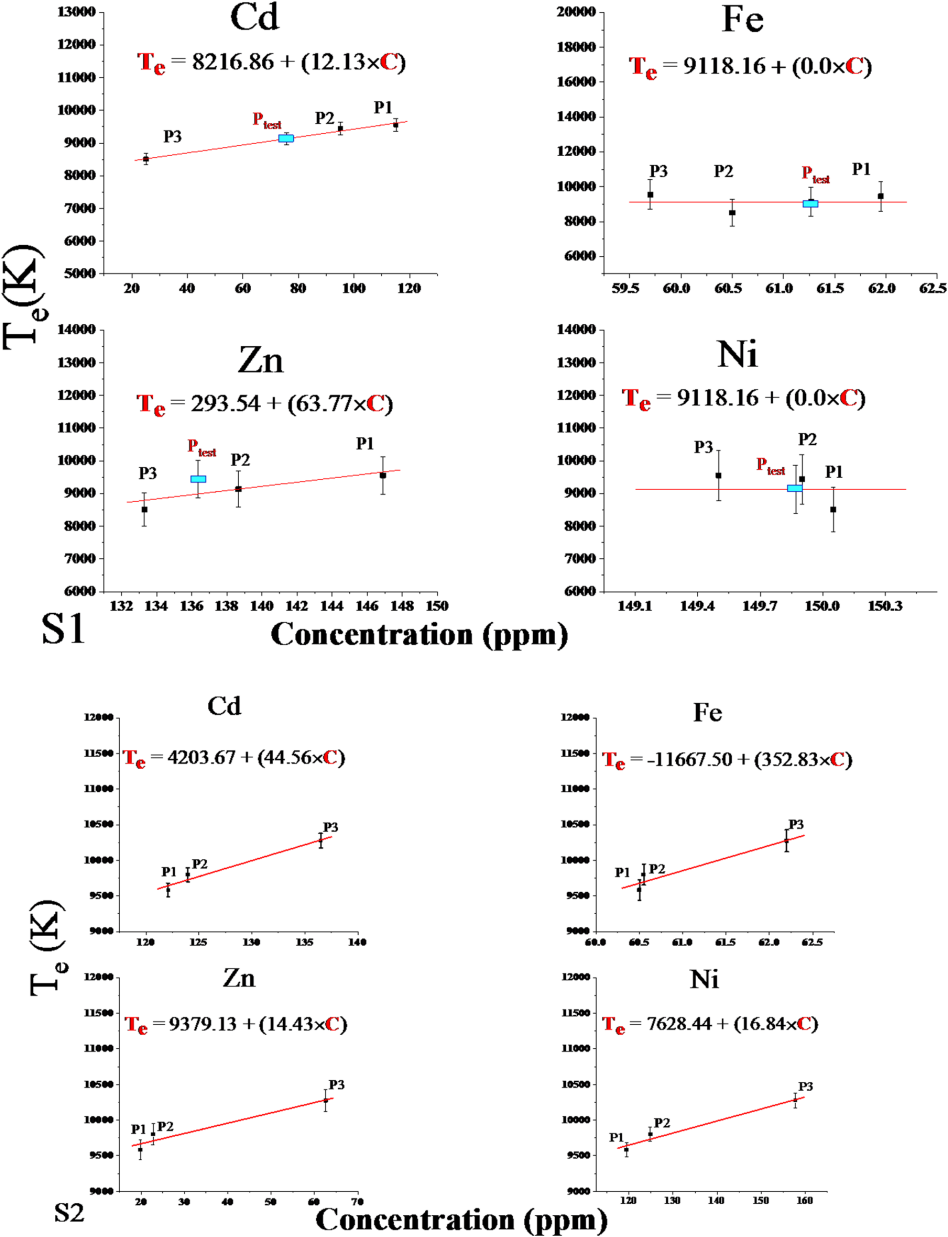
Calibration-free curves of Electron Temperature (T_e_) and Concentrations of contaminant elements for the two directions (S1) and (S2).

The linear fitting calibration-free curves of electron temperature and distance of all positions in the two directions are shown in Fig. 11. The observation and analysis of Fig. 11 show that T_e_ varied from 9556.9 to 8508.6 ± 1.5% and 9582.9 to10275.3 ± 0.5% for S1 and S2 respectively. These trends verify that the dependence of the electron temperature on the distance and site direction from the source of pollution is related to variations in the sample mass ablation rate and concentration of elements matrix in the sample, as shown in Fig. 12.

The corresponding experimental formulas resulted from Figs. 11 and 12 for determining the electron temperature gradient with distance (Eqs. 7 and 8) and elements concentration in the sample matrix (Table 4) for both directions S1 (Opposite to the wind direction) and S2 (With wind direction) improve the rules of the wind direction represent to the pollutant source as follow, where D is the distance from the factory (Eq. 6).

6 $$\:{\mathbf{T}}_{\mathbf{e}}\:\left(K\right)=\text{a}\:+\:\left(\text{b}\times\:\text{D}\left(\text{m}\right)\right).$$

Where T_e_ is the electron temperature, D is the distance from the fertilizer facility, and (a, and b) are empirically derived constants.

7 $$\:{{\mathbf{T}}_{\mathbf{e}}\:\left(\text{K}\right)}_{Opposite\:\:wind}=(\:9772.4\:-\:(\:0.616\times\:\:\text{D}\left(\text{m}\right))$$

8 $$\:{{\mathbf{T}}_{\mathbf{e}}\:\left(\text{K}\right)}_{With\:\:wind}=(\:9508.4\:+\:(0.384\times\:\:\text{D}\left(\text{m}\right)).$$

Table 3 illustrates the empirical formula of the electron temperature (T_e_) in kelvins for different elements (Cd, Fe, Zn, and Ni) in two directions (S1 and S2). The values of T_e_ are given as linear functions of the concentration (C) of the element. The table can be used to compare the electron temperatures of different elements in the same or other directions.

**Table 5:** The experimental empirical formula for calculating plasma electron temperature is based on contaminant elements’s concentrations.

Element	T_e_ (K), C (ppm)	
	S1	S2
Cd	T_e_ = 8216.86 + (12.13×C)	T_e_ = 4203.67 + (44.56×C)
Fe	T_e_ = 9118.16 + (0.0 × C)	T_e_ = – 11667.50 + (352.83×C)
Zn	T_e_ = 293.54 + (63.77×C)	T_e_ = 9379.13 + (14.43×C)
Ni	T_e_ = 9118.16 + (0.0×C)	T_e_ = 7628.44 + (16.84×C)

#### Validity confirmed through real-world sample analysis

The CF-Ps-LIPS developed method in the current study has shown promising potential for determining the concentrations of trace elements in complex samples. This study investigates the effectiveness of the CF-Ps-LIPS technique in quantifying four trace elements commonly found in environmental samples: cadmium (Cd), iron (Fe), zinc (Zn), and nickel (Ni). The estimated concentrations obtained through the CF-Ps-LIPS method were compared with those determined by Inductively Coupled Plasma-Optical Emission Spectrometry (ICP-OES), a well-established analytical technique. CF-Ps-LIPS quantified contaminant elements with ± 1% deviation from ICP-OES, outperforming traditional LIBS methods (± 5–10% error, Kumar et al., 2021)^43^. Plasma diagnostics revealed Neand T_e_ gradients (1.2–1.5 × 10^17^ cm^− 3^ and 8508–10,275 K) correlating with wind-driven contamination patterns (Figs. 8 and 9). These results mirror Fayek et al. (2025)^24^who linked N_e_ = 0.9–2.6 × 10^17^ cm^− 3^ to Cr/Pb levels in plants, validating CF-Ps-LIPS across environmental matrices.

The concentrations of the four trace elements in the test sample (P_test_) were determined using the CF-Ps-LIPS method through electron density and temperature.

Electron Density Approach: The Stark-broadening profile of the Ca I line at 646.257 nm was used as a standard reference line to calculate the electron density, as shown in Fig. 7. By substituting the obtained values of full-width at half-maximum (FWHM) and the Stark-broadening parameter (Ws) into the fitting Eq. (2), the electron density in the test sample was found to be 1.54382 ± 3.5% × 10^17^ cm^− 3^ (Fig. 8). The empirical formula from Table 4, using the later electron density value, yielded estimated concentrations in the P_test_ sample: 65.02 ppm Cd, 61.27 ppm Fe, 136.94 ppm Zn, and 149.87 ppm Ni^48^.

Electron Temperature Approach: The Boltzmann plots of Ca I lines at wavelengths 428.936, 442.544, 527.027, 585.745, and 612.222 nm were used to determine the electron temperature which agrees with previous investigations^49^. Figure 10 shows the slope of the Boltzmann plot, where ln(I.λ/Aki.gu) is plotted against the upper energy level Eu. The electron temperature was calculated to be 9136.3 K from the I.λ/Aki.gu value, as shown in Fig. 10. Using the electron temperature value in the empirical formula from Table 5 yielded estimated concentrations for the P_test_ sample: 75.80 ppm Cd, 61.27 ppm Fe, 138.66 ppm Zn, and 149.87 ppm Ni. Consequently, Table 6 summarizes the estimated concentrations of the four trace elements obtained from the CF-Ps-LIPS method using the associated values of electron density and electron temperature compared to the actual concentrations measured by ICP-OES. The results revealed a remarkable agreement, with deviations of only around ± 1% for all four elements. This close correlation between estimated and actual values validates the accuracy and effectiveness of the CF-Ps-LIPS method for real-world environmental samples, as demonstrated in previous studies^50,51^. The observed results confirm the validity of the developed CF-Ps-LIPS method using a real collected sample from the field and clarify its ability to accurately estimate the concentrations of trace elemental content without thoroughly analyzing the sample content, which aligns with the findings of Gaudiuso et al.^52^.

The CF-Ps-LIPS technique offers a significant advantage over conventional analytical methods by eliminating the need for time-consuming sample preparation and calibration procedures. This study demonstrates the potential of the CF-Ps-LIPS method as a rapid, reliable, and cost-effective tool for on-site monitoring and analysis of trace elements in various matrices, including environmental samples, industrial materials, and geological formations, consistent with the conclusions drawn by Pořízka et al. and Unnikrishnan et al.^53,54^.

**Table 6:** The concentrations of trace elements content in the test sample (Ptest) were determined using the CF-Ps-LIPS developed method, which considered plasma parameters fitting equations (plasma electron density and electron temperature) compared to the concentrations by ICP-OES.

Element	Concentration measured by ICP (ppm)	Unknown Concentration Calculations (S1)	
		*N*_e_	T_e_
Cd	69.62	65.02 ± 0.93%	75.80 ± 1%
Zn	145.33	136.94 ± 0.94%	138.66 ± 0.95%
Fe	61.67	61.27 ± 0.99%	61.27 ± 0.99%
Ni	149.53	149.87 ± 1%	149.87 ± 1%

## Conclusion

This study pioneers CF-Ps-LIPS as a paradigm shift in environmental monitoring, achieving calibration-free quantification of contaminant elements (Cd, Zn, Fe, Ni) in soils with ICP-OES-level precision. The method’s spatial resolution, enabled by 170 ps laser pulses and plasma diagnostics (N_e_, T_e_), addresses critical gaps in industrial contamination studies, particularly wind-driven dispersion. Building on Fayek et al. (2025)^24^we demonstrate CF-Ps-LIPS’s versatility across soil and plant matrices, positioning it as a cornerstone for rapid, on-site environmental safeguarding. The CF-Ps-LIPS method, validated using a test sample of unknown concentration, demonstrates its reliability and efficiency in real-world applications. Its versatility across various environmental matrices sets a new standard in the field, enabling rapid, precise quantifications for immediate on-site assessments and facilitating early pollution detection and timely remediation efforts. The potential development of portable CF-Ps-LIPS devices could revolutionize environmental safeguarding practices. This research presents a novel, calibration-free method for environmental analysis and underscores the critical role of innovative technologies in protecting ecosystems and public health, establishing a benchmark for future advancements in environmental sciences. Future work will expand validation to diverse soil types and integrate machine learning for real-time analysis.

## Data Availability

The data will be available upon request walid_tawfik@niles.edu.eg.
